# Antihypertensive and antihyperglycemic effects of combinations of losartan with metformin and/or glibenclamide in desoxycorticosterone acetate and streptozotocin-induced hypertensive diabetic rats

**DOI:** 10.1186/s42826-023-00159-2

**Published:** 2023-04-14

**Authors:** Emuesiri Goodies Moke, Eric Kelly Inanemo Omogbai, SammyDavies Ehiosu Osagie-Eweka, Adaeze Phina Uchendu, Odion Martha Obayuwana, Elizabeth Okoro-Akpandu, Benneth Ben-Azu

**Affiliations:** 1 Department of Pharmacology, Faculty of Basic Medical Sciences, Delta State University, Abraka, Nigeria; 2 Department of Pharmacology and Toxicology, Faculty of Pharmacy, University of Benin, Benin City, Nigeria; 3 Department of Biochemistry, Faculty of Life Sciences, University of Benin, Benin City, Nigeria; 4 Department of Physiology, School of Basic Medical Sciences, University of Benin, Benin City, Nigeria

**Keywords:** Desoxycorticosterone acetate, Diabetes, Hypertension, Losartan, Streptozotocin

## Abstract

**Background:**

Hypertension is a medical condition that often comorbidly exist in patients with type II diabetes. Therefore, it is very important to manage both conditions simultaneously to mitigate the complications and mortality connected with this comorbidity. Hence, this study investigated the antihypertensive and antihyperglycemic effects of combinations of losartan (LOS) with metformin (MET) and/or glibenclamide (GLB) in hypertensive diabetic rats. Hypertensive diabetic state was induced with desoxycorticosterone acetate (DOCA) and streptozotocin (STZ) in adult Wistar rats. The rats were divided into 5 groups (n = 5): control group (group 1), hypertensive diabetic (HD) control (group 2), treatment groups receiving LOS + MET (group 3), LOS + GLB (group 4), and LOS + MET + GLB (group 5). Group 1 comprised healthy rats while groups 2–5 were HD rats. The rats were treated orally once daily for 8 weeks. Fasted blood glucose (FBS) level, haemodynamic parameters, and some biochemical indices were thereafter assessed.

**Results:**

FBS level and blood pressure measurements were significantly (*P* < 0.05) increased following induction by DOCA/STZ. The drug treatment combinations, particularly combination of LOS + MET + GLB, significantly (*P* < 0.05) reduced the induced hyperglycemia and remarkably decreased systolic blood pressure and heart rate. There was significant (*P* < 0.05) reduction in raised lactate dehydrogenase and creatinine kinase levels by all drug treatment combinations except LOS + GLB.

**Conclusions:**

Our findings suggest that LOS combinations with MET and/or GLB exhibited significant antidiabetic and antihypertensive effects against DOCA/STZ-induced hypertensive diabetic state in rats.

## Background

Diabetes mellitus (DM) is a chronic endocrine and metabolic disorder that is characterized by elevated blood glucose level generally referred to as hyperglycemia and it is due to deficiency in insulin secretion or action [1, 2]. DM poses a global threat as millions of individuals are currently affected, with the figure set to double by 2030 [3, 4]. High morbidity (illness) and mortality (death) levels of diabetes amongst the Nigerian population have been reported [5, 6]. The disease may arise either as result of either the pancreatic β-cells does not produce enough insulin to regulate blood glucose level (Type I DM) or cells do not respond to the insulin produced (Type II DM) [7, 8]. DM is phenotypically characterized by high blood sugar, glucosuria and several microvascular and macrovascular complications linked to endocrine and metabolic dysfunctions [9]. Classical symptoms of the disease include frequent urination (polyuria), intense thirst (polydipsia) and hunger (polyphagia).

The pathogenesis of DM has been reported as multifactorial. There are some evidence largely suggesting the role oxidative damage, inflammation, apoptosis of pancreatic beta cells and prolonged increased levels vasopressin, which is released within the hypothalamic supraoptic nucleus [10, 11]. Long-term elevated levels of hyperglycemia are associated with numerous complications including hyperglycemia-induced microvascular damage [12]. This vascular damage has been linked to rigidification of vascular structures, notably mediated by excessive generation of free radicals and altered vascular immune system [12]. Remarkably, the complications of diabetes are linked to hyperglycemia-induced oxidative stress which may therefore overwhelms the body’s innate natural anti-oxidant system [13, 14]. This is significantly due to altered glucose, lipid and protein metabolisms as well as glycation of proteins as well as a vicious cycle of reactive oxygen species (ROS) generation in different body organs including the cardiovascular system [15, 16].

Indeed, hypertension is a major cardiovascular disease that poses health threat to both developed and developing countries with over 40% of cardiovascular mortality resulting from the interplay between genetic and environmental factors [17]. Hypertension is characterized of persistent increase in systolic blood pressure (SBP) above 140 mmHg or a diastolic blood pressure (DBP) above 90 mmHg [18]. It is classified into two categories: primary hypertension of which has no identifiable etiology and accounts for over 90% of cases, and secondary hypertension (10%) which is the elevation of blood pressure from identifiable causes including increased catecholamine, altered renin-angiotensin pathway, and increased sodium and water retention [19]. Comorbidity of DM and essential hypertension is very predominant and often linked to lowered life satisfaction of sufferers [20]. Patho-mechanistically, diabetes-induced exacerbated discharge of the sympathetic, renin-angiotensin aldosterone systems, as well as resistance to insulin, and increased insulin blood level (hyperinsulinemia), are predisposing pathological factors to hypertension [21–24]. Mounting bodies of evidence revealed that poorly managed comorbid DM and hypertension is an important promoting risk factor for the occurrence renal impairment and stroke among victims [21–23]. Given the multifaceted etiological factors mediating essential hypertension, we used deoxycorticosterone acetate (DOCA)-salt induced hypertension, which is a more translational, neurogenic and a high salt diet model of hypertension [25]. Among multiple mechanisms, DOCA-salt-induced hypertension was strongly linked to impaired renin-angiotensin pathway and hypervolemia, in which the kidney reabsorbs excessive sodium and water due to deranged renal sodium handling capacity [25]. Intriguingly, the combination of DOCA and streptozotocin (STZ), which is an established, popular preclinical animal model of DM, have been previously used to mimic a comorbid state of type 2 DM and hypertension which was characterized of decreased levels of blood insulin level and sustained high blood pressure. However, the etiological mechanisms remain incomplete [26] and moreover, no pharmacological treatment was applied on the animals.

In both comorbid cases, DM and hypertension can be managed by dietary modifications, exercise, and lifestyle changes [27]. With the use of hypoglycaemic drugs such as sulphonylureas, biguanides, thiazolidinediones, alpha-glucosidase inhibitors, and/or insulin, DM can be effectively mitigated [28, 29]. In hypertensive conditions, therapeutic agents used include diuretics, calcium channel blockers (CCBs), angiotensin-converting enzymes inhibitors (ACEIs), angiotensin II receptor blockers (ARBs), β-adrenoceptor blockers, amongst others [30]. Although simultaneous treatment of both conditions in hypertensive diabetic patients is intended to primarily attenuate the risk of macrovascular complications and mortality, there have been crosstalk on the different levels BP to be achieved in patients with DM [31, 32]. Thus, experts in the field recommend BP reduction of 130 mmHg and 80 mmHg for SBP and DBP respectively in patients with DM [32–34]. Accordingly, the Eighth Joint National Committee report of 2014, recommended that initial drug therapy for hypertension (including diabetic patients) should include a thiazide-type diuretic, a CCB, ACE inhibitor, or ARB (for non-black patients) and a thiazide-type diuretic, or a CCB for black patients [33, 34]. Also, a recent systematic review and network meta-analysis of 42 clinical trials, suggest that reducing SBP to between 120 and 124 mmHg with commonly used antihypertensive medications may result in a significant decline in the risk of cardiovascular disease and mortality [35]. A study was shown to demonstrate the effect of enalapril, an ACE inhibitor on a hypertensive-diabetic state induced by DOCA-STZ but without a combination with any antidiabetic agents, thereby limiting the scope of the outcomes [36]. Furthermore, investigation of the interactive effects of a commonly used antihypertensive drug such as losartan, and antidiabetic drugs including metformin and/or glibenclamide in hypertensive-diabetic-like conditions as simulated by DOCA-STZ in rats remain unknown. In line with this, we investigated the antidiabetic and antihypertensive effects of the combination of an ARB (losartan) with a biguanide (metformin) and/or a sulfonylurea (glibenclamide) in DOCA/STZ-induced hypertensive diabetic rats. This was to specifically elucidate the hemodynamics associated with the therapeutic interactions as well as characterized the mechanisms associated with tissue injuries.

## Results

### Effects of losartan, metformin, and glibenclamide on body weight of DOCA + STZ hypertensive diabetic rats

Table 1 shows the effects of LOS, MET and GLB on body weight of DOCA + STZ-induced hypertensive diabetic rats. There was significant (*P* < 0.05) decrease in the body weight of the HD control at week 4 and week 8 when compared with the normal control. Drug treatments with LOS + MET, LOS + GLB, and LOS + MET + GLB showed decrease in body weight throughout the treatment period, however, LOS + GLB and LOS + MET + GLB had significance (*P* < 0.05) across week 3 to week 8 when compared with the HD control.

**Table 1:** Effects of losartan, metformin, and glibenclamide on body weight of DOCA + STZ hypertensive diabetic rats

Treatment	Initial	Week 4	Week 8
Normal Control	237.20 ± 3.61	296.40 ± 6.20	317.80 ± 9.17
HD Control	273.60 ± 6.23	190.00 ± 6.12^*^	167.40 ± 4.45^*^
HD + LOS + MET	261.80 ± 7.55	214.60 ± 11.91^*^	200.40 ± 12.42^*^
HD + LOS + GLB	259.60 ± 10.79	250.20 ± 12.23^*#^	235.00 ± 13.78^*#^
HD + LOS + MET + GLB	265.00 ± 7.76	247.20 ± 1.50^*#^	225.80 ± 3.20^*#^

All values are expressed as mean ± SEM (n = 5). *P < 0.05 when compared with normal control; ^#^P < 0.05 when compared with hypertensive diabetic control. (*HD Control* Hypertensive Diabetic Control, *HD* Hypertensive Diabetic Rats, *LOS* Losartan, *MET* Metformin, *GLB* Glibenclamide)

### Effects of losartan, metformin and glibenclamide on 2-hourly fasting blood sugar level of DOCA + STZ hypertensive diabetic rats

Table 2 shows the effects of LOS, MET and GLB on fasting blood sugar level (hourly) of DOCA + STZ-induced hypertensive diabetic rats. There was a significant (*P* < 0.05) increase in FBS level of the HD control across the 2nd hour to 6th hour when compared with the normal control. Drug treatments with LOS + MET, LOS + GLB, and LOS + MET + GLB showed a significant (*P* < 0.05) decrease in FBS level across the 2nd hour to 6th hour when compared with the HD control.

**Table 2:** Effects of losartan, metformin and glibenclamide on 2-hourly fasting blood sugar level of DOCA + STZ hypertensive diabetic rats

Treatment	Post-induction (mg/dL)	2nd Hour	4th Hour	6th Hour
Normal Control	84.40 ± 2.69	76.40 ± 2.69	79.60 ± 2.20	72.80 ± 0.49
HD Control	354.60 ± 2.09	354.00 ± 1.79^*^	359.60 ± 3.36^*^	349.60 ± 1.83^*^
HD + LOS + MET	361.20 ± 7.84	290.40 ± 13.72^*#^	274.00 ± 8.57^*#^	256.00 ± 2.45^*#^
HD + LOS + GLB	329.60 ± 6.73	288.60 ± 3.80^*#^	246.80 ± 4.03^*#a^	225.20 ± 2.80^*#a^
HD + LOS + MET + GLB	382.00 ± 3.51	303.40 ± 7.45^*#^	261.40 ± 8.54^*#^	238.60 ± 5.67^*#a^

All values are expressed as mean ± SEM (n = 5). *P < 0.05 when compared with normal control; ^#^P < 0.05 when compared with hypertensive diabetic control; ^a^P < 0.05 when compared with LOS + MET treatment. (*HD Control* Hypertensive Diabetic Control, *HD* Hypertensive Diabetic Rats, *LOS* Losartan, *MET* Metformin, *GLB* Glibenclamide)

### Effects of losartan, metformin and glibenclamide on weekly fasting blood sugar level of DOCA + STZ hypertensive diabetic rats

Table 3 shows the effects of LOS, MET and GLB on fasting blood sugar level (weekly) of DOCA + STZ-induced hypertensive diabetic rats. There was a significant (*P* < 0.05) increase in FBS level of the HD control across week 1 to week 8 when compared with the normal control. Drug treatments with LOS + MET, LOS + GLB and LOS + MET + GLB showed significant (*P* < 0.05) decrease in FBS level across week 1 to week 8 when compared with the HD control.

**Table 3:** Effects of losartan, metformin and glibenclamide on weekly fasting blood sugar level of DOCA + STZ hypertensive diabetic rats

Treatment	Post-induction (mg/dL)	Week 1	Week 2	Week 3	Week 4	Week 8
Normal Control	84.40 ± 2.69	76.00 ± 1.26	78.60 ± 0.40	75.20 ± 1.66	75.60 ± 1.57	77.60 ± 3.26
HD Control	354.60 ± 2.09	358.00 ± 12.39^*^	357.40 ± 3.36^*^	340.20 ± 8.95^*^	333.60 ± 8.95^*^	301.80 ± 8.45^*^
HD + LOS + MET	361.20 ± 7.84	322.20 ± 6.85^*#^	291.40 ± 13.76^*#^	245.20 ± 6.47^*#^	234.80 ± 3.83^*#^	205.00 ± 8.44^*#^
HD + LOS + GLB	329.60 ± 6.73	294.20 ± 4.92^*#^	246.80 ± 8.50^*#a^	202.80 ± 3.14^*#a^	189.40 ± 8.51^*#a^	183.60 ± 9.36^*#^
HD + LOS + MET + GLB	382.00 ± 3.51	323.40 ± 10.06^*#^	276.80 ± 10.59^*#^	213.00 ± 3.00^*#a^	160.40 ± 9.04^*#a^	150.40 ± 8.21^*#a^

All values are expressed as mean ± SEM (n = 5). *P < 0.05 when compared with normal control; ^#^P < 0.05 when compared with hypertensive diabetic control; ^a^P < 0.05 when compared with LOS + MET treatment. (*HD Control* Hypertensive Diabetic Control, *HD* Hypertensive Diabetic Rats, *LOS* Losartan, *MET* Metformin, *GLB* Glibenclamide

### Effects of losartan, metformin and glibenclamide on systolic blood pressure of DOCA + STZ hypertensive diabetic rats

Table 4 shows the effects of LOS, MET and GLB on systolic blood pressure of DOCA + STZ-induced hypertensive diabetic rats. There was a significant (*P* < 0.05) increase in systolic BP of the HD control across week 1 to week 8 when compared with the normal control. Drug treatment with LOS + MET significantly (*P* < 0.05) decreased systolic BP at weeks 1, 4 and 8 when compared with the HD control. Whereas, treatments with LOS + GLB and LOS + MET + GLB showed significant (*P* < 0.05) decrease in systolic BP across week 1 to week 8 when compared with the HD control.

**Table 4:** Effects of losartan, metformin and glibenclamide on systolic blood pressure of DOCA + STZ hypertensive diabetic rats

Treatment	Initial (mmHg)	Week 1	Week 2	Week 3	Week 4	Week 8
Normal Control	89.67 ± 0.33	88.67 ± 2.73	89.33 ± 3.84	96.33 ± 7.33	97.33 ± 2.85	100.33 ± 2.73
HD Control	90.33 ± 3.18	127.67 ± 1.45^*^	134.33 ± 0.88^*^	147.67 ± 4.33^*^	147.67 ± 5.04^*^	146.67 ± 3.48^*^
HD + LOS + MET	93.33 ± 0.33	118.33 ± 1.76^*#^	126.67 ± 3.53^*^	130.67 ± 0.33^*^	120.67 ± 6.39^#^	115.67 ± 4.91^#^
HD + LOS + GLB	96.33 ± 0.33	118.67 ± 0.33^*#^	121.67 ± 1.20^*#^	128.33 ± 2.03^*#^	119.33 ± 6.36^#^	121.67 ± 2.03^*#^
HD + LOS + MET + GLB	95.00 ± 8.14	116.67 ± 1.76^*#^	118.33 ± 2.33^*#^	123.33 ± 0.88^*#^	111.33 ± 5.93^#^	112.00 ± 4.93^#^

All values are expressed as mean ± SEM (n = 5). *P < 0.05 when compared with normal control; ^#^P < 0.05 when compared with hypertensive diabetic control. (*HD Control* Hypertensive Diabetic Control, *HD* Hypertensive Diabetic Rats, *LOS* Losartan, *MET* Metformin, *GLB* Glibenclamide)

### Effects of losartan, metformin and glibenclamide on diastolic blood pressure (mmHg) of DOCA + STZ hypertensive diabetic rats

Table 5 shows the effects of LOS, MET and GLB on diastolic blood pressure of DOCA + STZ-induced hypertensive diabetic rats. There was a significant (*P* < 0.05) increase in diastolic BP of the HD control at weeks 1, 2, and 4 only when compared with the normal control. Drug treatments with LOS + MET and LOS + GLB showed significant (*P* < 0.05) decrease in diastolic BP only at week 4, while LOS + MET + GLB treatment significantly (*P* < 0.05) decrease in diastolic BP only at weeks 2 and 4 when compared with the HD control.

**Table 5:** Effects of losartan, metformin and glibenclamide on diastolic blood pressure of DOCA + STZ hypertensive diabetic rats

Treatment	Initial (mmHg)	Week 1	Week 2	Week 3	Week 4	Week 8
Normal Control	66.00 ± 0.00	59.67 ± 3.18	70.00 ± 8.62	75.00 ± 8.50	70.67 ± 4.33	80.00 ± 7.37
HD Control	64.00 ± 1.00	85.33 ± 3.71^*^	94.33 ± 0.67^*^	100.00 ± 1.00	106.00 ± 4.51^*^	80.33 ± 2.91
HD + LOS + MET	77.67 ± 3.76	79.33 ± 7.69	85.00 ± 1.53	87.67 ± 11.78	66.00 ± 1.15^#^	88.00 ± 6.25
HD + LOS + GLB	79.33 ± 3.71	74.33 ± 6.33	76.33 ± 0.67	69.67 ± 3.18	68.00 ± 7.02^#^	79.00 ± 0.00
HD + LOS + MET + GLB	78.67 ± 8.67	75.33 ± 5.33	72.67 ± 3.71^#^	77.33 ± 7.33	66.67 ± 6.67^#^	90.33 ± 2.33

All values are expressed as mean ± SEM (n = 5). *P < 0.05 when compared with normal control; ^#^P < 0.05 when compared with hypertensive diabetic control. (*HD Control* Hypertensive Diabetic Control, *HD* Hypertensive Diabetic Rats, *LOS* Losartan, *MET* Metformin, *GLB* Glibenclamide)

### Effects of losartan, metformin and glibenclamide on mean arterial pressure (mmHg) of DOCA + STZ hypertensive diabetic rats

Table 6 shows the effects of LOS, MET and GLB on mean arterial pressure of DOCA + STZ-induced hypertensive diabetic rats. There was significant (*P* < 0.05) increase in mean arterial pressure of the HD control across week 1 to week 4 when compared with the normal control. Drug treatments with LOS + MET showed significant (*P* < 0.05) decrease in mean arterial pressure at week 4 only, LOS + GLB at weeks 3 and 4 only while LOS + MET + GLB showed significant (*P* < 0.05) decrease in mean arterial pressure at weeks 2, 3, and 4 only when compared with the HD control.

**Table 6:** Effects of losartan, metformin and glibenclamide on mean arterial pressure (mmHg) of DOCA + STZ hypertensive diabetic rats

Treatment	Initial	Week 1	Week 2	Week 3	Week 4	Week 8
Normal Control	74.00 ± 0.00	69.00 ± 3.00	76.00 ± 7.02	82.00 ± 8.00	79.33 ± 3.28	86.33 ± 5.81
HD Control	72.33 ± 2.67	99.33 ± 0.67^*^	107.33 ± 3.71^*^	115.00 ± 2.89^*^	123.00 ± 6.25^*^	102.33 ± 1.33
HD + LOS + MET	81.67 ± 8.33	89.67 ± 89.67^*^	99.00 ± 2.08^*^	101.33 ± 0.67	84.33 ± 2.85^#^	97.00 ± 3.21
HD + LOS + GLB	83.33 ± 3.33	89.33 ± 0.33^*^	91.67 ± 1.67	89.00 ± 4.00^#^	85.33 ± 2.91^#^	93.00 ± 0.58
HD + LOS + MET + GLB	84.67 ± 2.67	89.00 ± 4.00^*^	88.00 ± 3.00^#^	93.67 ± 1.33^#^	82.67 ± 2.67^#^	94.00 ± 5.13

All values are expressed as mean ± SEM (n = 5). *P < 0.05 when compared with normal control; ^#^P < 0.05 when compared with hypertensive diabetic control. (*HD Control* Hypertensive Diabetic Control, *HD* Hypertensive Diabetic Rats, *LOS* Losartan, *MET* Metformin, *GLB* Glibenclamide)

### Effects of losartan, metformin and glibenclamide on heart rate (bpm) of DOCA + STZ hypertensive diabetic rats

Table 7 shows the effects of LOS, MET and GLB on heart rate of DOCA + STZ hypertensive-induced diabetic rats. There was a non-significant (*P* > 0.05) change in heart rate of the HD control across week 1 to week 8 when compared with the normal control. Drug treatments with LOS + MET, LOS + GLB and LOS + MET + GLB showed non-significant (*P* > 0.05) change in heart rate across week 1 to week 8 when compared with the HD control.

**Table 7:** Effects of losartan, metformin and glibenclamide on heart rate (bpm) of DOCA + STZ hypertensive diabetic rats

Treatment	Initial	Week 1	Week 2	Week 3	Week 4	Week 8
Normal Control	355.33 ± 18.48	330.00 ± 12.00	342.00 ± 12.17	342.67 ± 5.78	338.00 ± 6.51	318.67 ± 8.37
HD Control	351.33 ± 10.65	367.00 ± 10.07	374.00 ± 7.00	374.67 ± 4.10	380.00 ± 4.10^*^	381.67 ± 12.02^*^
HD + LOS + MET	396.00 ± 6.66	345.67 ± 14.67	327.00 ± 24.79	324.33 ± 6.67	314.33 ± 1.67^#^	315.67 ± 6.84^#^
HD + LOS + GLB	382.00 ± 13.80	371.33 ± 9.33	371.00 ± 2.00	353.00 ± 41.00	321.67 ± 12.35^#^	322.67 ± 0.88^#^
HD + LOS + MET + GLB	399.00 ± 9.45	340.33 ± 17.57	354.33 ± 12.78	324.33 ± 6.67	312.67 ± 1.67^#^	324.00 ± 4.93^#^

All values are expressed as mean ± SEM (n = 5). *P < 0.05 when compared with normal control; ^#^P < 0.05 when compared with hypertensive diabetic control. (*HD Control* Hypertensive Diabetic Control, *HD* Hypertensive Diabetic Rats, *LOS* Losartan, *MET* Metformin, *GLB* Glibenclamide)

### Effects of losartan, metformin and glibenclamide on cardiac injury markers and lipid profile of DOCA + STZ hypertensive diabetic rats

**Fig. 1:**
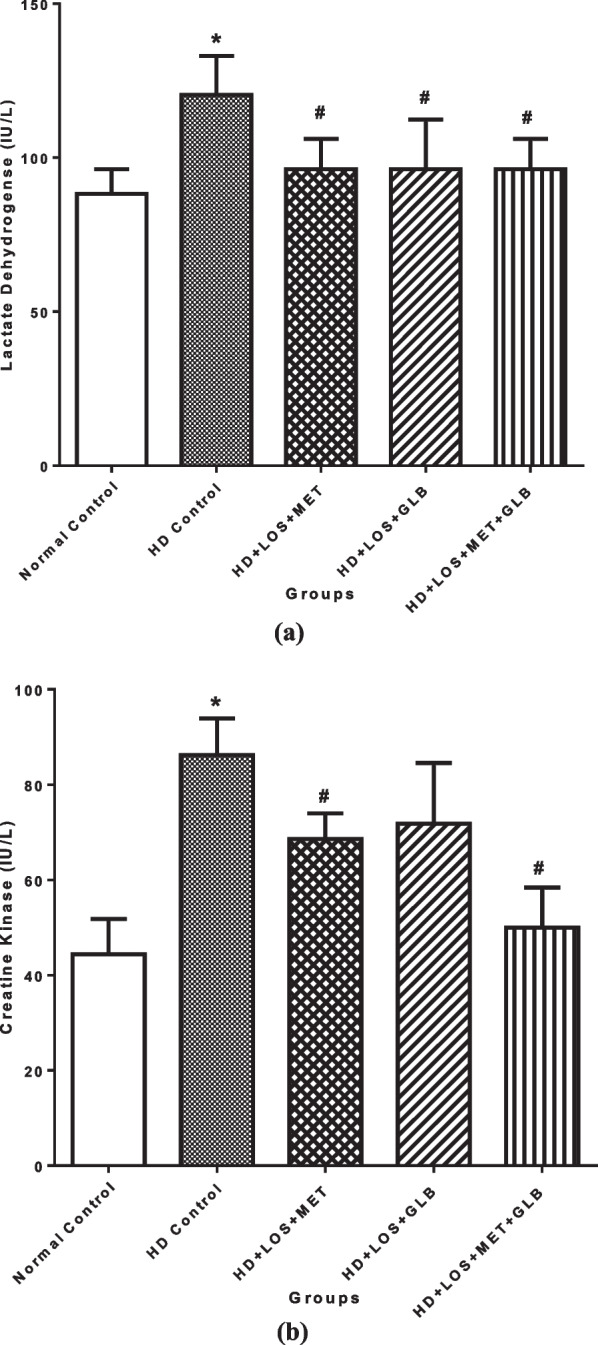
Effects of losartan, metformin and glibenclamide on lactate dehydrogenase (**a**) and creatine kinase (**b**) of DOCA + STZ hypertensive diabetic rats. Bars represent the mean ± SEM (n = 5). *P < 0.05 when compared with normal control; ^#^P < 0.05 when compared with hypertensive diabetic control. (*HD Control* Hypertensive Diabetic Control, *HD* Hypertensive Diabetic Rats, *LOS* Losartan, *MET* Metformin, *GLB* Glibenclamide)

The effects of LOS, MET and GLB on cardiac injury markers of DOCA + STZ-induced hypertensive diabetic rats is shown in Fig. 1. There was significant (*P* < 0.05) increase in LDH level of the HD control group when compared with the normal control. Drug treatments with LOS + MET, LOS + GLB and LOS + MET + GLB showed significant (*P* < 0.05) decrease in LDH when compared with the HD control. CK level was significantly (*P* < 0.05) increased in the HD control group when compared with the normal control. Treatments with LOS + MET, LOS + GLB and LOS + MET + GLB had significant (*P* < 0.05) decrease in CK when compared with the HD control.

**Fig. 2:**
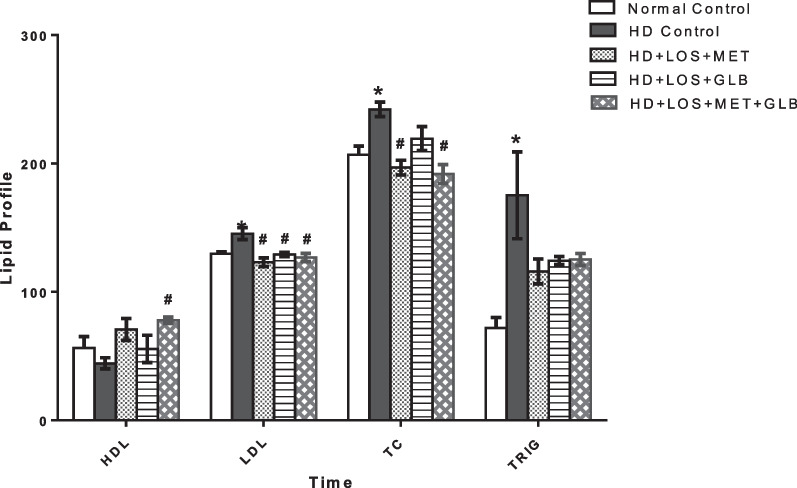
Effects of losartan, metformin and glibenclamide on lipid profile of DOCA + STZ hypertensive diabetic rats. Bars represent the mean ± SEM (n = 5). *P < 0.05 when compared with normal control; ^#^P < 0.05 when compared with hypertensive diabetic control. (*HD Control* Hypertensive Diabetic Control, *HD* Hypertensive Diabetic Rats, *LOS* Losartan, *MET* Metformin, *GLB* Glibenclamide)

Figure 2 shows the effects of LOS, MET and GLB on lipid profile of DOCA + STZ-induced hypertensive diabetic rats. There was significant (*P* < 0.05) decrease in HDL level of the HD control group when compared with the normal control. Drug treatments with LOS + MET, LOS + GLB and LOS + MET + GLB increased HDL, however, only LOS + MET + GLB treatment showed significant (*P* < 0.05) increase in HDL when compared with the HD control. LDL level was significantly (*P* < 0.05) increased in the HD control group when compared with the normal control. Treatments with LOS + MET, LOS + GLB and LOS + MET + GLB significantly (*P* < 0.05) decreased LDL when compared with the HD control. There was significant (*P* < 0.05) increase in total cholesterol level of the HD control group when compared with the normal control. Drug treatments with LOS + MET, LOS + GLB and LOS + MET + GLB showed significant (*P* < 0.05) decrease in total cholesterol when compared with the HD control. Triglyceride level was also significantly (*P* < 0.05) increased in the HD control group when compared with the normal control. Treatments with LOS + MET, LOS + GLB and LOS + MET + GLB non-significantly (*P* > 0.05) decreased triglyceride when compared with the HD control.

### Effects of losartan, metformin and glibenclamide on histology of pancreas and cardiac tissue of DOCA + STZ hypertensive diabetic rats

**Fig. 3:**
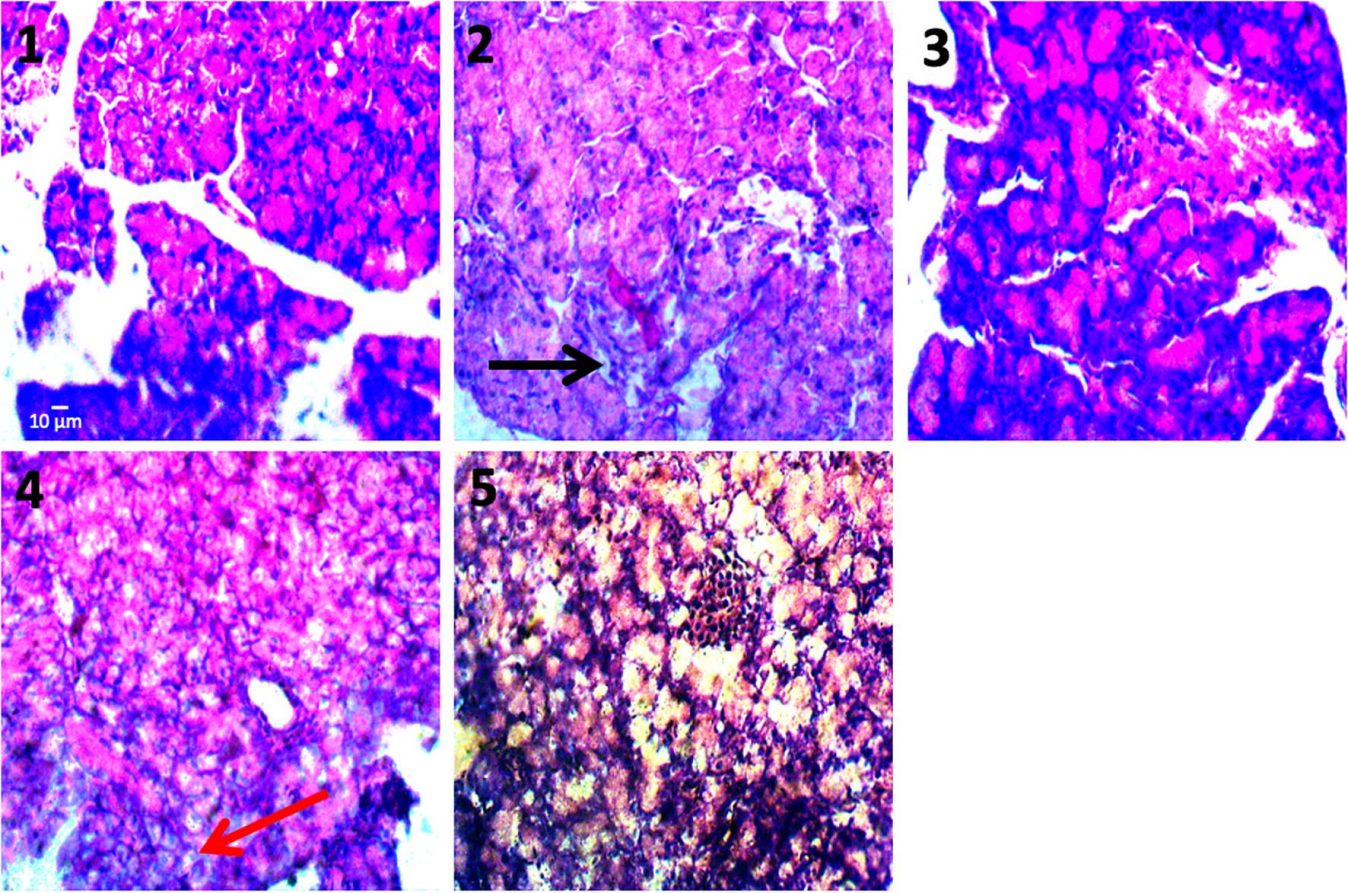
Effects of losartan, metformin and glibenclamide on the histology of the pancreas of DOCA+STZ hypertensive diabetic rats

**Fig. 4:**
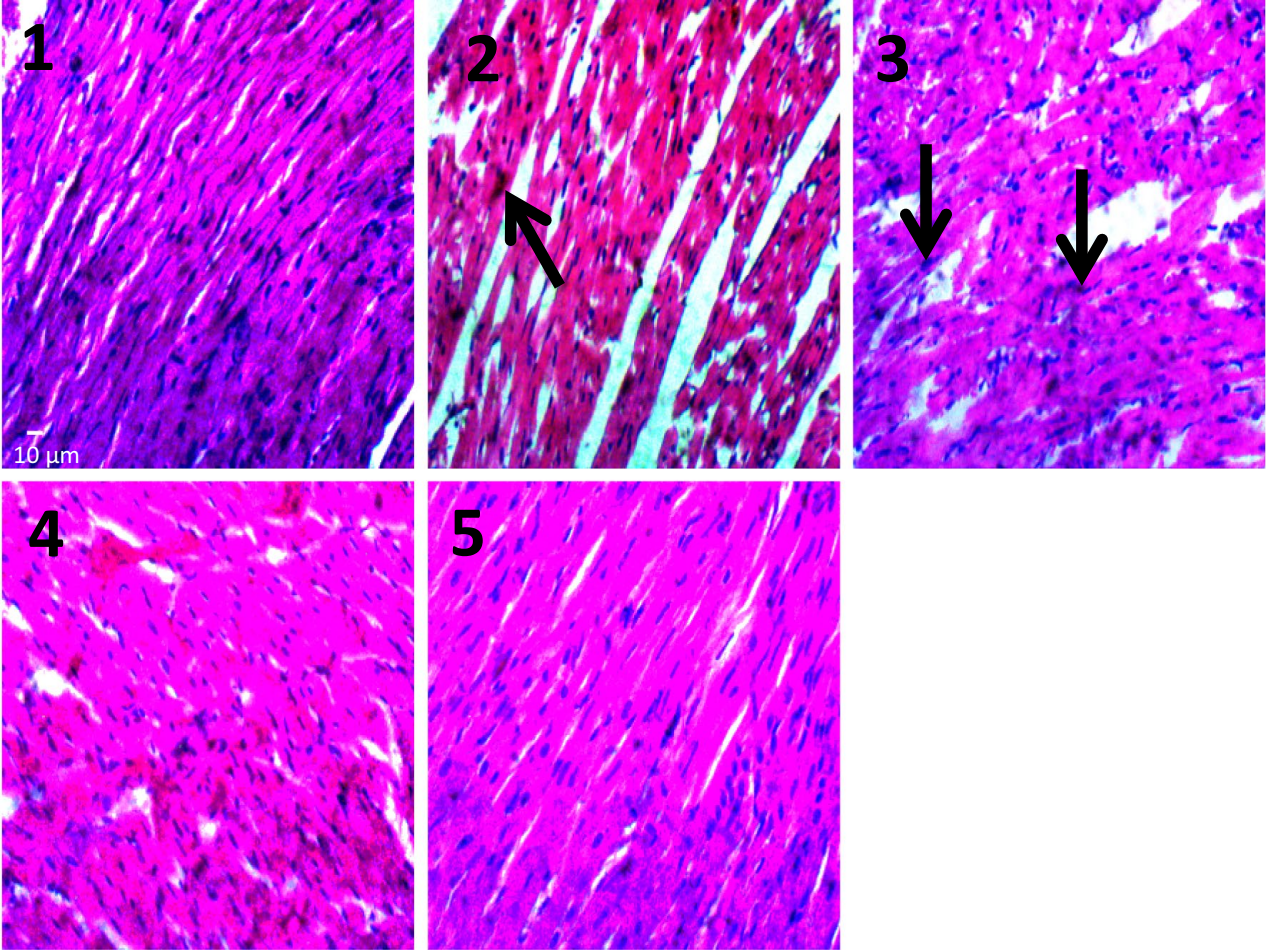
Effects of losartan, metformin and glibenclamide on the histology of the heart of DOCA+STZ hypertensive diabetic rats

The effects of LOS, MET and GLB on the histology of the pancreas of DOCA + STZ-induced hypertensive diabetic rats is shown in Fig. 3. Group 1 (normal control) shows no observable lesion of Islet cells; group 2 (HD control) shows severe necrosis and inflammation (black arrow) of the Islet cells; group 3 (HD + LOS + MET) shows necrosis and inflammation of Islet cells (red arrow); group 4 (HD + LOS + GLB) shows moderate atrophy of acinar and Islet cells; group 5 (HD + LOS + MET + GLB) shows no observable lesion of Islet cells. While on the histology of the heart of DOCA + STZ hypertensive diabetic rats (Fig. 4.), there was no observable lesion of myocardial cells with the normal control group; moderate atrophy and degeneration of myofibres (black arrow) with the HD control animals, moderate hypertrophy of myofibres (black arrows) in HD + LOS + MET group, and no observable lesion of myocardial cells in both HD + LOS + GLB and HD + LOS + MET + GLB groups.

## Discussion

The finding from this study showed that treatments with LOS + MET, LOS + GLB and LOS + MET + GLB significantly reduced DOCA/STZ-induced increased in body weight. Moreover, DOCA/STZ increased heart rate, systolic blood pressure as well as elevated blood sugar level when compared with controls. However, these changes were normalized by LOS + MET, LOS + GLB and LOS + MET + GLB treatments, respectively. Additionally, treatments with LOS + MET, LOS + GLB and LOS + MET + GLB significantly attenuated DOCA/STZ-induced increased LDH and CK concentrations. We also found that DOCA/STZ exposure caused a significant increase in LDL and total cholesterol levels, which were reduced by LOS + MET, LOS + GLB and LOS + MET + GLB relative to controls. However, treatments with LOS + MET, LOS + GLB and LOS + MET + GLB failed to mitigate DOCA/STZ-induced increased triglyceride levels in the rats relative to DOCA/STZ control. Excitingly, we also found that LOS + MET + GLB protected against DOCA/STZ-induced degeneration of the pancreatic beta cells and myocardial cells of the heart in comparison with DOCA/STZ groups respectively.

The DOCA-salt-induced hypertension is a translational neurogenic hypertensive model characterized of human cardiovascular remodeling due to impairment of renin-angiotensin pathway and hypervolemia, in which the kidney reabsorbs excessive sodium and water due to deranged renal sodium handling capacity [25]. It raises blood pressure through oxidative stress and impairment of renal function by increasing mineralocorticoid with subsequent increase in nicotinamide adenine dinucleotide phosphate (NADPH) oxidase activity and superoxide production [37, 38]. DOCA increases aldosterone to increase reabsorption of sodium ions and water from the epithelial cells in the distal nephron of the kidney to raise blood pressure [39].

DOCA/STZ significantly decrease body weight of HD control rats by 38.8% which is an indication that it interferes with the normal body growth of rats. Drug treatments significantly inhibited the reduction of body weight by 9.5% in LOS + GLB and 14.8% in LOS + MET + GLB but not in LOS + MET. Combinations with GLB had increase in body weight. This revealed that GLB, like every other sulfonylurea antidiabetic agent, is associated with weight gain [40].

Streptozotocin is a well-known diabetogenic agent [41]. Untreated hyperglycemia has been reported to induce numerous severe life-threatening complications that include injury to the eye, kidneys, nerves, heart, as well destruction of peripheral vascular system [21–23]. Thus, simultaneous treatment of hypertension and diabetes is intended to primarily attenuate the risk of macrovascular complications and mortality [32–34]. Moreover, a meta-analysis of 42 clinical trials, suggest that reducing SBP to between 120–124 mmHg with commonly used antihypertensive medications may result in a significant decline in the risk of cardiovascular disease and mortality [35]. In this study, we found that FBS level of HD control rats were significantly increased by DOCA/STZ to cause diabetes. All drug treatment combinations significantly reduced the FBS level at 2 h following single dose administration and for the 8 weeks period. These data revealed that the various drug treatment combinations possess effective antidiabetic effect on hypertensive diabetic conditions.

According to the American Diabetes Association (ADA) Standards of Medical Care in Diabetes 2019 update, sulfonylureas such as glyburide (glibenclamide) are considered one of the six options for adjunct therapy with MET, the first-line anti-diabetic medication [42, 43]. While MET has an onset of action of about 1.5 h, GLB possesses a rapid onset of 15–60 min [42]. Thus, combination treatment with metformin and sulfonylurea have been adjudged as an effective approach than when these drugs are applied singly to improving glycemic control in type 2 diabetes. Notably, Alotaibi and colleagues reported the therapeutic effectiveness of combining GLB with LOS in diabetic states [44]. The efficacy of the combination of antidiabetic agents have also been reported [45–48]. A separate study revealed the hypoglycemic activity of a combination of glimepiride (a sulfonyurea) and MET was enhanced when LOS was co-administered as a single dosage schedule as well as a multiple dose schedule [49].

Blood pressure parameters (SBP, DBP, MAP and HR) were significantly increased in DOCA/STZ rats which was possibly due to hypercholesteremia and oxidative stress-derived vasoconstriction in the animals. In this study, we observed that weekly administration of these drugs led to a dramatic reduction in SBP and HR, although there were no significance changes in DBP and MAP. Of note, it is an indication that the drug combinations can be used for effective management of blood pressure in hypertensive diabetic states, particularly by reducing systolic pressure [45].

As regards the estimation of biomarkers cardiac injury, serum levels of LDH and CK were used as previous described [50]. Accordingly, we also found that DOCA/STZ-induced comorbid hypertensive diabetic states were accompanied by significantly elevated serum levels of LDH and CK, which possibly suggest evidence of cardiac tissue damage [45, 50]. However, LOS + MET, LOS + GLB and LOS + MET + GLB produced a significant reduction in LDH level. Additionally, LOS + MET and LOS + MET + GLB, but not LOS + GLB markedly attenuated DOCA/STZ-induced elevated level of CK, which suggests that metformin combinations (LOS + MET and LOS + MET + GLB) produced a more protective effect against tissue injury.

Previous investigations have shown that dyslipidemia increases the risk of development of coronary artery disease and progression of atherosclerotic lesions [51, 52]. DOCA/STZ-induced hypertensive diabetes also precipitated hyperlipidemia evidenced by increased serum levels of total cholesterol, triglyceride, and LDL with reduced HDL level, a pathological mechanism we believed could in part, be responsible for the vascular rigidity damage that promoted hypertensive state. Interestingly, we found that combinational treatment with these drugs significantly reduced raised LDL caused by DOCA/STZ exposure. Herein, treatments with LOS + MET and LOS + MET + GLB were found to decrease the total cholesterol significantly, while no significant effect was seen on triglyceride levels. However, the HDL cholesterol level was solely improved significantly with all three combination (LOS + MET + GLB), suggesting that LOS + MET + GLB possesses a much more positive cardiovascular protective effects on hypertensive diabetic rats based on the hypolipidemic activity.

Histopathological results showed that hypertensive diabetic state induced by DOCA/STZ profoundly generated tissue damage in the pancreas and heart. This is depicted by the severe necrosis and inflammation of the Islet cells of the pancreas, and hypertrophy of myocardial cells with interstitial macrophage infiltration, evidenced by elevated levels of FBS and HBP parameters respectively. However, several studies have hypothesized that the clinical effects of antidiabetic and antihypertensive agents are based on their ability to repair or prevention damage to Islet cells of the pancreas and hypertrophy of myocardial cells. In this study, the combinational approach therapy with LOS + MET + GLB significantly reduced the DOCA/STZ-induced damages to the pancreas and heart tissues, suggesting pancreatic and myocadiac protective properties. However, marked attenuating effect on tissue damage was evident in LOS combination with both MET and GLB (LOS + MET + GLB) than when the antidiabetics are used singly with LOS.

## Conclusions

The findings from this study suggest that the combination of losartan, metformin and glibenclamide mitigate DOCA/STZ-induced comorbid states involving hypertension and diabetes via mechanisms associated with decreased fasting blood glucose level, systolic blood pressure and reduced markers of cardiac injury with corresponding decrease in body weight. The study recommends that for effective management of diabetes and hypertension comorbidity, both metformin and glibenclamide in combination with losartan may be a superior approach to prevent complications associated with this comorbidity.

## Methods

### Experimental animals

Healthy adult male Wistar rats aged 12–15 weeks and weighing between 220 and 280 g were randomly selected for the study. Animals were obtained from the Animal Facility of the Department of Pharmacology, Ambrose Alli University, Ekpoma, Edo State, Nigeria. The animals were acclimatized for 14 days prior to the study, and were fed with standard animal pellets (Chikun Feed® Grower Pellet, Nigeria) and clean water ad libitum. Guidelines followed in the handling of animals were in accordance with the ethical standards of the ‘National Institute of Health Guide for the Care and Use of Laboratory Animals’ as adopted by the ethical committee of the Faculty of Pharmacy, University of Benin, Benin City, Nigeria. Ethical approval was obtained prior to the commencement of the experiment (Ethical number – EC/FP/018/01).

### Drugs and chemicals

Streptozotocin (STZ) (sc-200719; Santa Cruz Biotechnology, Dallas, TX, USA), deoxycorticosterone acetate (DOCA) (sc-239659; Santa Cruz Biotechnology), losartan potassium (sc-204796A; Santa Cruz Biotechnology), metformin (sc-202000B; Santa Cruz Biotechnology), glibenclamide (sc-200982A; Santa Cruz Biotechnology) and sodium chloride (NaCl) (LOBA Chemie PVT Ltd, India) were used in this study.

### Induction of diabetes and hypertension

Type 2 diabetes was induced with a combination of high-fat diet and a single intraperitoneal injection of streptozotocin (STZ; 45 mg/kg) in sterile citrate buffer (0.1 M, pH 4.5) to fasted male Wistar rats [53]. It is paramount to induce type 2 diabetes mellitus as the mechanisms of hypoglycaemic action of metformin and glibenclamide involves available beta cells [54]. Diabetic state of the rats was checked after 72 h by means of a glucometer (ACCU-CHEK® Active) and compatible blood glucose test strips, and animals with hyperglycaemia of fasting blood glucose level of ≥ 200 mg/dl were selected for the study [55, 56].

The DOCA salt induced hypertension model was used to mimic secondary form of hypertension. Method of uninephrectomy (UNX) was done according to Hemalatha et al., [57]. Briefly, rats kidney were visualized by a left lateral abdominal incision (1 cm long) while the left renal artery and ureter were tied by a silk thread, and DOCA-salt (25 mg/kg) was administered a week after for 8 weeks [58].

### Experimental design

Group 1: Normal + distilled water (10 ml/kg) (Normal control)

Group 2: DOCA + STZ + distilled water (10 ml/kg) (HD control)

Group 3: DOCA + STZ + LOS + MET

Group 4: DOCA + STZ + LOS + GLB

Group 5: DOCA + STZ + LOS + MET + GLB

[*HD* Hypertensive diabetic; *LOS* Losartan (50 mg/kg); *MET* Metformin (500 mg/kg); *GLB* glibenclamide (5 mg/kg)]

Drugs were administered orally daily for a period of eight (8) weeks with a weekly measurement of fasting blood sugar (FBS) and blood pressure (BP).

### Measurement of body weight

Body weight of animals were measured weekly in grams to estimate the effect of the induced diabetes and hypertension on body composition. The weight was measured using a digital electronic weighing balance.

### Measurement of fasting blood sugar level

Fasting blood sugar (FBS) level was measured using a glucometer (ACCU-CHEK® Active) with compatible blood glucose test strips. FBS level at 2nd, 4th and 6th hours following single drug administration was measured. Afterwards, weekly measurements of FBS for 8 weeks were taken.

### Measurement of blood pressure

Blood pressure measurements (systolic blood pressure—SBP, diastolic blood pressure—DBP, mean arterial pressure—MAP and heart rate—HR) were recorded in the conscious rats weekly during daylight (between 8 am and 12 noon) by the same investigator, using a tail-cuff plethysmography (MRBP system, IITC Life Science, Woodland Hills, CA, USA), a computerized non-invasive blood pressure system which measures rat’s tail blood pressure by means of volume pressure. The rat was positioned in the animal holder (restrainer) with necessary adjustments made to ensure a restricted animal movement, and leaving the tail outside the holder. The restrainer was then placed in the heating chamber and heated up to 32℃. The basic software setup of the system was calibrated prior to start of measurement. The BP monitoring sensor was placed round the tail root of rats. Following animal movement stability, the data of SBP, DBP, MAP and HR were recorded [45].

### Measurements of cardiac injury markers and lipid profile

Serum cardiac injury markers including creatine kinase (CK) and lactase dehydrogenase (LDH) were assessed spectrophotometrically according to standard manufacturer’s procedure as seen in the assay kits (Sigma- Aldrich, Germany). High density lipoprotein (HDL) and triglyceride (TG) were evaluated using the assay kit’s procedures (Elabscience, USA) and (Randox, England) respectively. Serum total cholesterol was determined using the method described by Trinder [59], while low density lipoprotein (LDL) was calculated according to Friedewald et al. [60].

### Histology of pancreas and cardiac tissue

Tissue sections of the pancreas and the left ventricle of the heart from all the groups were processed for histological examination according to procedures described by Kieman [61].

### Statistical analysis

All data obtained were expressed as Mean ± SEM (standard error of mean), and analyzed by one-way analysis of variance (ANOVA) followed by Tukey’s post hoc test. Analysis was done using GraphPad Prism version 7.0 (GraphPad Software, San Diego, CA). P-values < 0.05 were taken as significant. Data were presented in tables and graphs.

## Data Availability

All data and materials are available upon request.

## Abbreviations

ACE: Angiotensin-converting enzyme
ACEI: Angiotensin-converting enzyme inhibitor
ADA: American Diabetes Association
ANOVA: One-way analysis of variance
ARB: Angiotensin II receptor blocker
BP: Blood pressure
CCB: Calcium channel blockers
CK: Creatine kinase
DBP: Diastolic blood pressure
DM: Diabetes mellitus
DOCA: Deoxycorticosterone acetate
FBS: Fasting blood sugar
GLB: Glibenclamide
HD: Hypertensive diabetic
HDL: Low-density lipoprotein
HR: Heart rate
JNC: Joint National Committee
LDH: Lactate dehydrogenase
LDL: High-density lipoprotein
LOS: Losartan
MAP: Mean arterial pressure
MET: Metformin
NADPH: Nicotinamide adenine dinucleotide phosphate
SBP: Systolic blood pressure
SEM: Standard error of mean
STZ: Streptozotocin
UNX: Uninephrectomized
WHO: World Health Organization

## References

[1] Karalliedde J, Gnudi L (2016). Diabetes mellitus, a complex and heterogeneous disease, and the role of insulin resistance as a determinant of diabetic kidney disease. Nephrol Dial Transplant.

[2] Enhörning S, Melander O (2018). The vasopressin system in the risk of diabetes and cardiorenal disease, and hydration as a potential lifestyle intervention. Ann Nutr Metab.

[3] Saeedi P, Petersohn I, Salpea P, Malanda B, Karuranga S, Unwin N, et al. IDF Diabetes Atlas Committee. Global and regional diabetes prevalence estimates for 2019 and projections for 2030 and 2045: Results from the International Diabetes Federation Diabetes Atlas, 9th edition. Diabetes Res Clin Pract. 2019;157:107843. 10.1016/j.diabres.2019.107843 31518657

[4] Khan MAB, Hashim MJ, King JK, Govender RD, Mustafa H, Al KJ (2020). Epidemiology of type 2 diabetes—global burden of disease and forecasted trends. J Epidemiol Glob Health.

[5] Agofure O, Odjimogho S, Okandeji-Barry OR, Efegbere HA, Nathan HT (2020). Pattern of diabetes mellitus-related complications and mortality rate: Implications for diabetes care in a low-resource setting. Sahel Med J.

[6] Adeloye D, Ige JO, Aderemi AV, Adeleye N, Amoo EO, Auta A (2017). Estimating the prevalence, hospitalisation and mortality from type 2 diabetes mellitus in Nigeria: a systematic review and meta-analysis. BMJ Open.

[7] Fu Z, Gilbert ER, Liu D (2013). Regulation of insulin synthesis and secretion and pancreatic Beta-cell dysfunction in diabetes. Curr Diabetes Rev.

[8] American Diabetes Association (ADA). Diagnosis and classification of diabetes mellitus. Diabetes Care. 2014;37(Suppl 1):81–90. 10.2337/dc14-S081 24357215

[9] Naserrudin NA, Jeffree MS, Kaur N, Syed Abdul Rahim SS, Ibrahim MY. Diabetic retinopathy among type 2 diabetes mellitus patients in Sabah primary health clinics-Addressing the underlying factors. PLoS One. 2022;17:e0261249. 10.1371/journal.pone.0261249 PMC8797256 35089931

[10] Al Doghaither H, Elmorsy E, Al-Ghafari A, Ghulam J (2021). Roles of oxidative stress, apoptosis, and inflammation in metal-induced dysfunction of beta pancreatic cells isolated from CD1 mice. Saudi J Biol Sci.

[11] Oh YS, Bae GD, Baek DJ, Park EY, Jun HS (2018). Fatty acid-induced lipotoxicity in pancreatic beta-cells during development of type 2 diabetes. Front Endocrinol (Lausanne).

[12] Giri B, Dey S, Das T, Sarkar M, Banerjee J, Dash SK (2018). Chronic hyperglycemia mediated physiological alteration and metabolic distortion leads to organ dysfunction, infection, cancer progression and other pathophysiological consequences: an update on glucose toxicity. Biomed Pharmacother.

[13] Fiorentino TV, Prioletta A, Zuo P, Folli F (2013). Hyperglycemia-induced oxidative stress and its role in diabetes mellitus related cardiovascular diseases. Curr Pharm Des.

[14] Sharifi-Rad M, Anil Kumar NV, Zucca P, Varoni EM, Dini L, Panzarini E (2020). Lifestyle, oxidative stress, and antioxidants: back and forth in the pathophysiology of chronic diseases. Front Physiol.

[15] American Diabetes Association (ADA). Diagnosis and classification of diabetes mellitus. Diabetes Care. 2010;33 (Suppl 1):62–9. 10.2337/dc10-S062 PMC2797383 20042775

[16] Matough FA, Budin SB, Hamid ZA, Alwahaibi N, Mohamed J (2012). The role of oxidative stress and antioxidants in diabetic complications. Sultan Qaboos Uni Med J.

[17] Guo X, Zou L, Zhang X, Zheng L, Sun Z (2011). Prehypertension: A metaanalysis of the epidemiology, risk factors, and predictors of progression. Tex Heart Inst J.

[18] Mills KT, Stefanescu A, He J (2020). The global epidemiology of hypertension. Nat Rev Nephrol.

[19] Monesha G, Ashish B, Sanjay S, Syed H, John T, Michelle B (2015). Essential hypertension vs. Secondary hypertension among children. Am J Hypertens.

[20] Petrie JR, Guzik TJ, Touyz RM (2018). Diabetes, hypertension, and cardiovascular disease: clinical insights and vascular mechanisms. Can J Cardiol.

[21] Einarson TR, Acs A, Ludwig C, Panton UH (2018). Prevalence of cardiovascular disease in type 2 diabetes: a systematic literature review of scientific evidence from across the world in 2007–2017. Cardiovasc Diabetol.

[22] Leon BM, Maddox TM (2015). Diabetes and cardiovascular disease: Epidemiology, biological mechanisms, treatment recommendations and future research. World J Diabetes.

[23] Lastra G, Syed S, Kurukulasuriya LR, Manrique C, Sowers JR (2014). Type 2 diabetes mellitus and hypertension: an update. Endocrinol Metab Clin North Am.

[24] Ramalingam L, Menikdiwela K, LeMieux M, Dufour JM, Kaur G, Kalupahana N (2017). The renin angiotensin system, oxidative stress and mitochondrial function in obesity and insulin resistance. Biochim Biophys Acta Mol Basis Dis.

[25] Lin HY, Lee YT, Chan YW, Tse G (2016). Animal models for the study of primary and secondary hypertension in humans (review). Biomed Rep.

[26] Dai S, Fraser H, McNeill JH (1992). Effects of deoxycorticosterone acetate on glucose metabolism in nondiabetic and streptozotocin-diabetic rats. Can J Physiol Pharmacol.

[27] Agbonifo-Chijiokwu E, Nwangwa KE, Oyovwi MO, Ben-Azu B, Naiho AO, Emojevwe V (2023). Underlying biochemical effects of intermittent fasting, exercise and honey on streptozotocin-induced liver damage in rats. J Diabetes Metab Disord.

[28] American Diabetes Association Professional Practice Committee; Draznin B, Aroda VR, Bakris G, Benson G, Brown FM, Freeman R, et al. Pharmacologic approaches to glycemic treatment: Standards of medical care in diabetes-2022. Diabetes Care. 2022;45 Suppl 1:125–43. 10.2337/dc22-S009 34964831

[29] Tsapas A, Avgerinos I, Karagiannis T, Malandris K, Manolopoulos A, Andreadis P (2020). Comparative effectiveness of glucose-lowering drugs for type 2 diabetes: a systematic review and network meta-analysis. Ann Intern Med.

[30] Feldman RD, Hussain Y, Kuyper LM, McAlister FA, Padwal RS, Tobe SW (2015). Intraclass differences among antihypertensive drugs. Annu Rev Pharmacol Toxicol.

[31] Grossman A, Grossman E (2017). Blood pressure control in type 2 diabetic patients. Cardiovasc Diabetol.

[32] Cosentino F, Grant PJ, Aboyans V, Bailey CJ, Ceriello A, Delgado V (2020). 2019 ESC Guidelines on diabetes, pre-diabetes, and cardiovascular diseases developed in collaboration with the EASD. Eur Heart J.

[33] James PA, Oparil S, Carter BL, Cushman WC, Dennison-Himmelfarb C, Handler J (2014). 2014 evidence-based guideline for the management of high blood pressure in adults: report from the panel members appointed to the Eighth Joint National Committee (JNC 8). JAMA.

[34] Unger T, Borghi C, Charchar F, Khan NA, Poulter NR, Prabhakaran D (2020). 2020 International society of hypertension global hypertension practice guidelines. Hypertension.

[35] Bundy JD, Li C, Stuchlik P, Bu X, Kelly TN, Mills KT (2017). Systolic blood pressure reduction and risk of cardiovascular disease and mortality: a systematic review and network meta-analysis. JAMA Cardiol.

[36] Goyal RK, Satia MC, Bangaru RA, Gandhi TP (1998). Effect of long-term treatment with enalapril in streptozotocin diabetic and DOCA hypertensive rats. J Cardiovasc Pharmacol.

[37] Seifi B, Kadkhodaee M, Karimian SM, Zahmatkesh M, Xu J, Soleimani M (2010). Evaluation of renal oxidative stress in the development of DOCA-salt-induced hypertension and its renal damage. Clin Exp Hypertens.

[38] Araujo M, Wilcox CS (2014). Oxidative stress in hypertension: role of the kidney. Antioxid Redox Signal.

[39] Lefebvre H, Duparc C, Naccache A, Lopez AG, Castanet M, Louiset E (2019). Paracrine regulation of aldosterone secretion in physiological and pathophysiological conditions. Vitam Horm.

[40] Chaudhury A, Duvoor C, Reddy Dendi VS, Kraleti S, Chada A, Ravilla R (2017). Clinical review of antidiabetic drugs: implications for type 2 diabetes mellitus management. Front Endocrinol (Lausanne).

[41] Lenzen S (2008). The mechanisms of alloxan- and streptozotocin-induced diabetes. Diabetologia.

[42] Ho J, Leung AKC, Rabi D (2011). Hypoglycaemic agent in the management of type 2 diabetes mellitus. Recent Pat Endocr Metab Immune Drug Discov.

[43] American Diabetes Association (ADA). Pharmacologic approaches to glycemic treatment: Standards of medical care in diabetes-2019. Diabetes Care. 2019;42 Suppl 1:90–102. 10.2337/dc19-S009 30559235

[44] Alotaibi MR, Fatani AJ, Almnaizel AT, Ahmed MM, Abuohashish HM, Al-Rejaie SS (2019). *In vivo* assessment of combined effects of glibenclamide and losartan in diabetic rats. Med Princ Pract.

[45] Moke EG, Omogbai EKI, Osagie-Eweka SDE, Uchendu AP, Omogbiya AI, Ben-Azu B (2023). Co-administration of metformin and/or glibenclamide with losartan reverse N^G^-nitro-l-arginine-methyl ester-streptozotocin-induced hypertensive diabetes and haemodynamic sequelae in rats. Microvasc Res.

[46] Tosi F, Muggeo M, Brun E, Spiazzi G, Perobelli L, Zanolin E (2003). Combination treatment with metformin and glibenclamide versus single-drug therapies in type 2 diabetes mellitus: a randomized, double-blind, comparative study. Metabolism.

[47] Lamos EM, Stein SA, Davis SN (2012). Combination of glibenclamide-metformin HCl for the treatment of type 2 diabetes mellitus. Expert Opin Pharmacother.

[48] Nachum Z, Zafran N, Salim R, Hissin N, Hasanein J, Gam Ze Letova Y (2017). Glyburide versus metformin and their combination for the treatment of gestational diabetes mellitus: A randomized controlled study. Diabetes Care.

[49] Nagaraju B, Anilkumar KV (2021). Pharmacodynamic and pharmacokinetic interaction of losartan with glimepiride-metformin combination in rats and rabbits. Indian J Pharmacol.

[50] Kristjansson RP, Oddsson A, Helgason H, Sveinbjornsson G, Arnadottir GA, Jensson BO (2016). Common and rare variants associating with serum levels of creatine kinase and lactate dehydrogenase. Nat Commun.

[51] Ariyanti R, Besral B (2019). Dyslipidemia associated with hypertension increases the risks for coronary heart disease: a case-control study in Harapan Kita Hospital, National Cardiovascular Center. Jakarta J Lipids.

[52] Osagie-Eweka SDE, Orhue NEJ, Omogbai EKI, Moke EG (2022). Methanol leaf extract of *Simarouba glauca* induced dyslipidemia-linked cardiovascular disease indicators and its effect on antioxidant proteins. Scientia Africana.

[53] Furman BL (2021). Streptozotocin-induced diabetic models in mice and rats. Curr Protoc.

[54] Foretz M, Guigas B, Bertrand L, Pollak M, Viollet B (2014). Metformin: from mechanisms of action to therapies. Cell Metab.

[55] Asiwe JN, Anachuna KK, Moke EG, Sanusi KO, Okonofua DE, Omeru O (2021). High dietary salt intake alleviates fasting blood glucose in streptozotocin-induced diabetic male Wistar rats. Thai J Pharm Sci.

[56] Okonofua DE, Asiwe JN, Anachuna KK, Moke EG, Sanusi KO, Adagbada EO (2021). Effect of diabetes mellitus and hypertension on osmotic fragility and hemorheological factors in male wistar rats. Biol Med Natural Prod Chem.

[57] Hemalatha G, Pugalendi KV, Saravanan R (2013). Modulatory effect of sesamol on DOCA-salt-induced oxidative stress in uninephrectomized hypertensive rats. Mol Cell Biochem.

[58] Edosuyi O, Choi M, Igbe I, Oyekan A (2021). Fumarate exerted an antihypertensive effect and reduced kidney injury molecule (KIM)-1 expression in deoxycorticosterone acetate-salt hypertension. Clin Exp Hypertens.

[59] Trinder P (1969). Determination of blood glucose using an oxidase-peroxidase system with a non-carcinogenic chromogen. Ann Clin Biochem.

[60] Friedewald WT, Levy RI, Fredrickson DS (1972). Estimation of the concentration of low-density lipoprotein cholesterol in plasma, without use of the preparative centrifuge. Clin Chem.

[61] Kieman JK (1990). Histology and Histological Methods, Theory and Practice.

